# Beyond ‘The Squeaky Wheel’: The Passion, Precarity and Potential of the Family/Carer Lived Experience Workforce in the Queensland Mental Health System

**DOI:** 10.1111/hex.70720

**Published:** 2026-06-08

**Authors:** Katherine Reid, Lorna Downes, Victoria Stewart, Kath Sellick, Marianne Wyder

**Affiliations:** ^1^ Queensland University of Technology, School of Public Health and Social Work, Kelvin Grove Campus QLD Australia; ^2^ School of Health Sciences and Social Work Griffith University Logan Campus Queensland Australia; ^3^ Department of Social Work University of Melbourne Melbourne Victoria Australia; ^4^ Metro South Addiction and Mental Health Services Queensland Australia

**Keywords:** family inclusion and relational recovery, family peer work, family/carer lived experience workforce, mental health services

## Abstract

**Background:**

Although family inclusion is embraced in mental health policy and practice guidelines, families continue to report feeling devalued and/or excluded from service delivery. Family/Carer Lived Experience (LE) workers play an important role in supporting and advocating for families with a member who is accessing a mental health service. While embedding lived experience in service provision is a priority, there is limited understanding of the role of the Family/Carer LE workforce.

**Method:**

Using a survey and focus groups, this mixed methods study investigated workforce conditions and available supports for Family/Carer LE workers employed in the Queensland mental health system, Australia.

**Results:**

Key findings demonstrated that this workforce is passionate about working relationally with families yet, face precarious workplace conditions including lack of role clarity, lack of job security, prioritisation of bio‐medical approaches over LE expertise, and experiences of moral injury. Findings illustrated how Family/Carer LE workers' experiences can mirror their carer experience, with many reporting being isolated and unsupported in their workplace roles.

**Discussion:**

These work conditions impact on the Family/Carer LE workforce and undermine the sustainability of their roles. Recommendations point to the need for organisational readiness, role clarity, adequate support, training and supervision, and meaningful integration into mental health services.

**Conclusion:**

This study underscores the need for systemic change to ensure family inclusion becomes a reality as opposed to rhetoric. Until such time Family/Carer LE workers will continue to be ‘the squeaky wheel’.

**Patient or Public Contribution:**

The research team consisted of four academics experienced in family/carer inclusion and/or with Family/Carer LE, and two Family/Carer LE leaders (one(one of which was a researcher). A Family/Carer LE Worker Advisory Group comprising seven Family/Carer LE workers guided each stage of the research process, particularly adapting the workforce survey. A Family/Carer LE researcher from our team was involved in the study's conceptualisation, data collection, data analysis and preparation of the manuscript.

## 1 Introduction

### 1.1 Family Inclusion in Mental Health Service Delivery

Family inclusion in mental health care is embedded in policies, and organisational guidelines nationally and internationally, with carers and family acknowledged as integral partners in the care of individuals experiencing mental distress [1, 2, 3]. Despite this recognition, many families continue to report feeling excluded from mental health care processes [4, 5], while having to manage significant risk [6]. Families continue to occupy an ambiguous space, often defined as the ‘burdened carer’ or seen as contributing to, or causing distress for their family member [7]. Families and carers are rarely recognised in their own right [8]; Wyder, Jonas & Bland, 2025). Mental health systems largely remain focused on individualised care models [9] which fail to account for the significant role that families, friends and carers play in a person's recovery journey [10] and their own recovery journeys [6]; Wyder, Jonas & Bland, 2025).

In this paper, the term ‘consumer' is used to describe a person with lived experience of mental distress, and ‘carer' to refer to a family member, partner, friend or other person who provides informal, unpaid support. Preferences for terminology vary across contexts, however, these terms are used here for consistency.

Relational recovery approaches emphasise the importance of interpersonal relationships in the healing process and highlight the reciprocal nature of recovery for both individuals and their support networks (Bland & Drake, 2019 [10, 11, 12];). While families and carers frequently provide critical emotional and practical assistance [13] they can also experience significant psychological and social strain due to their caregiving responsibilities [14]. These experiences, combined with the stigma associated with mental illness, can lead to family members and carers experiencing social exclusion and isolation [15] and burn out [6]. Many families report receiving inadequate support from mental health services [6, 16]. As a result, families are often expected to provide ongoing support while being excluded from key decision‐making processes [17, 18, 19]).

### 1.2 Embedding Lived Experience in Service Delivery

In recent years, mental health services have increasingly recognised the value of integrating LE into their service delivery models [20] employing LE or peer workers, whose responsibilities include direct support, advocacy, and system change work through the lens of LE [21]. While traditionally this workforce was comprised of people who have experienced mental health challenges (consumers), there is a growing inclusion of people with LE of providing informal, unpaid care to family or friends accessing mental health services [22].

Research on consumer LE roles identifies persistent challenges including organisational culture, role ambiguity, and misconceptions about peer work [23]. Additional concerns include limited recognition from clinical staff, who may not view these roles as professionally legitimate [24], as well as experiences of inequity, and discrimination [25]. These workplace conditions can expose the LE workforce to morally distressing and or injurious events [26] such as witnessing consumers, and their families and carers being treated oppressively. These events violate the LE worker's ‘deeply held beliefs and expectations about wrong and right’ and can causes psychological harm [27], p. 1).

While some of these issues may relate to the Family/Carer LE workforce, there remains a notable lack of research regarding the specific challenges encountered by Family/Carer LE workers [28, 29]. Importantly, the experiences of families and carers differ from those of consumers, resulting in distinct values, objectives, and practice approaches (Roennfeldt et al., 2023). Common barriers for the Family/Carer LE workforce include inadequate organisational support [30], and service cultures that devalue relational approaches and marginalise their contributions [31].

In Australia, Family/Carer LE roles within the mental health system have evolved from strong traditions of family and carer advocacy, supported by national and state policy directives promoting consumer and carer participation [32]. Current guidelines emphasise the integration of these roles to advance recovery‐oriented, person‐centred care, with workers drawing on lived experiences of supporting someone through mental health challenges to foster empowerment and hope [33]. While the importance of these roles has been recognised for many years, workforce development and organisational support remain limited.

Although policy frameworks endorse family‐inclusive practices, their application across Queensland's mental health services remains inconsistent. There is limited research investigating the impacts of this lack of structure on the Family/Carer LE workforce. To address this gap, in 2021 and 2022 the Rising Together project through a co‐design process conducted a mixed‐methods participatory research project into the experiences of the Family/Carer LE workers (Sellick et al., 2024). The authors observed that despite their dedication, Family/Carer LE workers in Victoria, Australia, often felt isolated, undervalued, and faced inconsistent workplace conditions and support (Sellick et al., 2024). This is despite, to the best of our knowledge, Victoria being the only Australian state that has an established Carer Lived Experience Workforce (CLEW) network to support this workforce. Many other jurisdictions, including Queensland, lack formalised systems to recognise and support Family/Carer LE roles. The absence of these support structures raises concerns not only about the long‐term viability of this workforce but also its potential to drive meaningful system change. To explore the experiences of the Family/Carer LE workers within the Queensland mental health system, the current study was guided by two central questions: ‘What are the experiences of the Family/Carer LE workforce in the Queensland mental health system?’ and ‘What factors support safe and effective workplaces for the Family/Carer LE workers in Queensland?’

## 2 Methods

### 2.1 Design

This research adopted a mixed‐methods approach to examine the experiences and workplace conditions of Family/Carer LE workers within Queensland's mental health system. Mixed methods approaches are often used in health care research to enable a comprehensive investigation of the research question, by identifying patterns across the quantitative data and further nuance and contextualised insights from qualitative data [34]. This research design enabled a breadth of findings and at the same time, depth to understanding Family/Carer LE workforce experiences. Ethical clearance for the study was granted by the University's Human Research Ethics Committee (Ref No: 2023/866). The research was conducted in two phases.

### 2.2 Phase 1: Online Survey

The first phase involved an online survey gathering both quantitative and qualitative data. The survey instrument (Suppl 1) was adapted from the *Rising Together* study (Sellick et al., 2024) to reflect Queensland's specific context. These adaptations were guided by an Advisory Group composed of seven Family/Carer LE workers from urban and regional Queensland. Based on their input, additional questions were added to explore workplace safety and moral injury. The final survey included questions regarding workforce demographics, workplace conditions, role‐specific concerns, workplace supports such as supervision, and opportunities for professional growth and career development. The survey had open‐ended, closed, and Likert‐scale items. The survey was disseminated through carer advocacy organisations, other mental health networks and social media platforms between June and August 2024. A diverse range of non‐government and government mental health services operating in urban, regional, and remote areas in Queensland were also contacted via email or phone to confirm the existence of Family/Carer LE roles. This workforce mapping exercise enabled a wider distribution of the survey.

### 2.3 Phase 2: Focus Groups

The final phase involved conducting two online focus groups to investigate the themes identified in the survey data, one in December 2024 (with 12 participants) and another in January 2025 (with 8 participants). These sessions aimed to provide rich, qualitative insights into participants' workplace experiences. Survey findings were shared with participants, followed by open‐ended prompts to encourage reflection and discussion. Topics explored included organisational culture, safety, role definition, access to training and supervision, career pathways, and workforce sustainability. The 2‐h session followed a semi‐structured format and was recorded and transcribed. Transcriptions were de‐identified by a member of the research team. Participants were also asked to reply to some questions via the chat function, which were also included in the data.

### 2.4 Data Analysis

Quantitative data from the survey were analysed using Microsoft Excel and SPSS (Version 30). Descriptive statistics, including frequencies, means, and medians, were used to summarise demographic characteristics and workplace experiences. The thematic analysis of the qualitative data from open‐ended survey responses and focus group transcripts were guided by Braun and Clarke's (2016) six‐phase approach. Three researchers (KR, VS and LD) independently familiarised themselves with the data and generated initial codes. Codes and coding discrepancies were resolved through discussion with the wider research team and grouped into broader categories to identify concepts and overarching themes. The final codes were then re‐applied to the full data set. The qualitative data was managed in NVivo (Version 14).

### 2.5 Findings

The findings from the survey and focus groups are presented together to provide a cohesive and in‐depth picture of the Family/Carer LE workforce in Queensland. The findings offer a demographic overview of the research participants and explore the three key themes identified. These themes include: the passion of the Family/Carer LE workforce, the precarious workplace conditions and ways to support the potential of the workforce. These will be outlined below.

#### 2.5.1 Demographic Overview

A total of 37 individuals completed the Family/Carer LE survey, representing approximately 40% of the estimated workforce identified during an initial mapping phase. The majority of respondents identified as female (92%), with over half aged 50 or older (51.3%). Participants reported a diverse range of caring experiences, including support for children under 18 (51.4%), adult children (35.1%), partners or spouses (40.5%), siblings (43.2%), parents (35.1%), and friends (18.9%). Multiple responses were permitted.

In terms of diversity, 10.8% of respondents identified as Aboriginal or Torres Strait Islander, 10.8% identified as LGBTQI + , and 8.1% reported living with a disability. Notably, 37.8% of participants disclosed experiencing psychological distress themselves, and 62.2% indicated that they were also consumers of mental health services. Educationally, a significant portion of the workforce (69.7%) held more than one formal qualification. Further demographic details are outlined in Table 1.

**Table 1:** Participant details.

Characteristic		*n*	%
Age (years)	18–29	3	8.1
	30–39	5	13.5
	40–49	10	27.1
	50–59	13	35.1
	60–69	6	16.2
Highest level of education	Secondary school	2	5.4
	Vocational education certificate/diploma	16	43.3
	Undergraduate tertiary qualification	10	27.0
	Postgraduate tertiary qualification	9	24.3
Length of time working in the role	Less than a year	8	21.6
	1–2 years	8	21.6
	3–5 years	11	29.7
	6–10 years	6	16.2
	Over 10 years	4	10.8
Location of employment	Metropolitan	19	51.4
	Rural (> 10,000 people)	9	24.3
	Remote (< 10,000 people)	2	5.4
	Statewide	5	13.5
	National	2	5.4
Employment area*	Child & Youth mental health	19	51.4
	Adult mental health	17	45.9
	Alcohol & other drugs	6	16.2
	Older persons mental health	10	27.0
Employment hours	Full time	11	29.7
	Part time	22	59.5
	Casual	4	10.8

* Participants could select all that applied.

#### 2.5.2 The Passion of the Family/Carer LE Workforce

Both the quantitative and qualitative data highlighted that Family/Carer LE workers were committed, passionate and believed they were making a difference. Most survey respondents (83.8%) believed that they made a difference to the families and carers they work with, and 78.3% believed they were valued by families and carers. Additionally, 75.6% agreed or strongly agreed they were ‘satisfied with the work that I do as a Family/Carer Lived Experience worker’.

Discussions from the focus groups emphasised this passion. Working relationally to provide vital assistance, supporting service navigation, advocacy, and focusing on the families own recovery were strong themes. The quote below illustrates this passion.

The thing that I enjoy the most would be that connection, absolutely, but it's also when I was going through the Child and Youth Mental Health system, I felt absolutely lost. I was isolated, I was floundering, I didn't know what was happening. I was completely overwhelmed, and there was no one there who was able to support me or to explain and help navigate the mental health system. It was quite devastating.

[FGP5]

#### 2.5.3 The Importance of Working Relationally

Results indicated the effectiveness of the Family/Carer LE approach stems from its relational foundation, which prioritises amplifying the voices of families. Central to this, is peer‐to‐peer engagement, enabling genuine connection with carers and families through shared lived experience and mutual understanding. The significance of working relationally was explained in following quotes.

Peer work comes from a relational, mutual and reciprocal understanding of connection.

[FGP6]

Most importantly, what I love is the fact that there's no barrier between any conversation that I may find myself involved in, because when I'm at that level, I'm the same ‐ there's no expertise, I'm the same, so it's a different conversation. That's what I love.

[FGP7]

Relational support for families and carers consistently involved creating ‘a really safe, supportive, non‐judgemental space’ [FGP5]. This nurturing environment aimed to alleviate stress and ensure carers had the ‘energy and time and space for their caring role’ [FGP5]. Peer‐to‐peer interactions were considered vital, not only for supporting families but also for redirecting attention toward carers' own wellbeing. These exchanges helped carers acknowledge their personal recovery journeys, which may differ from those of the individuals they support. This perspective was captured in the following reflection:

When it's a carer‐to‐carer… those conversations flow you know, it helps to help feel validated, but also I'm able to help them consider their own caring needs and their own recovery goals that can be separate to the person that they're caring for.

[FGP11]

Additionally, relational work involved assisting families in navigating complex systems and linking them with appropriate services was a shared priority among the Family/Carer LE workforce. As one participant expressed:

I like being able to help people navigate and be able to find services easier than some of our past experiences.

[FGP9]

#### 2.5.4 Passion for Advocacy

Several Family/Carer LE workers spoke about their passion for advocacy, with, and on behalf of families and carers. This advocacy was across a range of levels including influencing service delivery, organisational culture and policy, as well, as supporting families and carers to build their self‐advocacy skills. The following quotes illustrate this point.

I like being that squeaky wheel and speaking up for families when they're not able to speak for themselves.

[FGP11]

Empowering the carers and parents to sort of be able to communicate with clinicians or systems going forward, beyond our service, I guess, reminding them that they're the expert in their young person and not to be afraid to voice their opinion in a respectful, productive way.

[FGP5]

Participants embraced the role of being the ‘squeaky wheel’ in the system if this meant families and carers experienced better service responses.

#### 2.5.5 Precarious Workplace Conditions

Although Family/Carer LE workers expressed strong commitment to their roles, both survey findings and focus group discussions focused on the precarity of their employment conditions. The quantitative data showed that over half (59.5%) were working part‐time and 24.3% of respondents indicated a desire to increase their working hours. Permanent employment was uncommon, with just 21.6% reporting that their position was permanent.

Concerns around equitable pay were common, with 27% of the survey participants agreeing that there were disparities in employment conditions compared to colleagues in non‐lived experience roles. Only 37.8% of survey participants felt their pay was fair.

Flexibility in work arrangements was also limited, with only 18.9% stating they had ‘access to flexible working arrangements due to my caring role.’ This pay disparity and lack of flexible hours were discussed in the focus group conversations. One Family/Carer LE worker remarked that the ‘hours of work [were] very inflexible’ [FGP5]. Other participants shared:

I also think that the pay is not fair in that we're often stretched across the whole [service] … whereas clinicians are working, within a team…we're working across the full breadth of all the families.

[FGP10]

I made the conscious decision to go into carer peer work and not continue on the journey with social work. But I can tell you the workload is very, very comparable and I don't really understand why that's not reflected in the pay scales.

[FGP10]

These work conditions serve to place the Family/Carer LE workforce in a precarious position.

#### 2.5.6 Lack of Role Clarity and Challenges in Working in Multi‐Disciplinary Teams

Family/Carer LE workers commonly spoke about a lack of role clarity, with some workers not having a position description. Participants highlighted how the lack of role understanding negatively impacted on how Family/Carer LE workers were perceived by the rest of the team. The quotes below exemplify these experiences.

There is overall confusion on our scope of work, the purpose of lived experience within the team (sic).

[Survey respondent]

When a multidisciplinary team doesn't understand my role, there is mistrust, conflict, judgement and competition.

[FGP5]

Even when a role description was provided, most participants reported changing expectations and being asked to perform tasks beyond their defined responsibilities. Participants also noted a lack of guiding policies or practice frameworks, which perpetuated this lack of role clarity.

There are no clear guidelines on the Carer/Family peer worker role that has been provided from the LE team. The role I have entered feels responsive to whatever the team feels they need support with regarding a family and this has often veered into Social Work skills, child safety etc… yet we are not paid accordingly for these skills.

[Survey respondent]

This lack of role clarity perpetuated the Family/Carer LE workforce precarious position in their organisations.

Although survey respondents reported that their Family/Carer LE worker roles were mostly valued within organisations (56.7%, with 29.7% strongly agreeing and 32.4 agreeing – see Table 2), this did not necessarily translate to an understanding and support of the Family/Carer LE roles by all colleagues. While there were high levels of understanding, valuing and support from other Family/Carer LE colleagues, there was less understanding, valuing and support received from non‐lived experience colleagues (see Table 2).

**Table 2:** Support from colleagues.

Statement	Strongly agree	Agree	Neutral	Disagree	Strongly disagree
*Support from Family/carer LE colleagues*					
I am understood	12 (32.4%)	18 (48.6%)	4 (10.8%)	3 (8.1%)	0
I am valued	12 (32.4%)	18 (48.6%)	5 (13.5%)	2 (5.4%)	0
I am supported	14 (37.8%)	16 (43.2%)	4 (10.8%)	2 (5.4%)	1 (2.7%)
*Support from consumer LE colleagues*					
I am understood*	5 (13.5%)	18 (48.6%)	9 (24.3%)	1 (2.7%)	0
I am valued*	7 (18.9%)	18 (48.6%)	7 (18.9%)	1 (2.7%)	0
I am supported*	5 (13.5%)	20 (54.1%)	7 (18.9%)	1 (2.7%)	0
*Support from non‐LE colleagues (e.g., nurses)*					
I am understood	2 (5.4%)	16 (43.2%)	13 (35.1%)	5 (13.5%)	1 (2.7%)
I am valued	6 (16.2%)	15 (40.5%)	12 (32.4%)	3 (8.1%)	1 (2.7%)
I am supported	6 (16.2%)	17 (45.9%)	11 (29.7%)	3 (8.1%)	0
*Support from my organisation*					
My role is understood*	5 (13.5%)	13 (35.1%)	11 (29.7%)	4 (10.8%)	3 (8.1%)
My role is valued	11 (29.7%)	12 (32.4%)	6 (16.2%)	4 (10.8%)	4 (10.8%)
I am supported	9 (24.3%)	13 (35.1%)	8 (21.6%)	1 (2.7%)	6 (16.2%)

* Not all participants responded to this question.

These mixed levels of support within the organisation are reflected in the finding that only 40.5 per cent of participants reporting not feeling excluded in the workplace due to their LE role (Table 3). These survey results demonstrate that not all participants feel safe in the workplace, consolidating a precarious employment position.

**Table 3:** Organisational safety.

Statement	Strongly agree	Agree	Neutral	Disagree	Strongly disagree
I feel excluded in the workplace	5 (13.5%)	5 (13.5%)	11 (29.7%)	10 (27.0%)	5 (13.5%)
I am free to express my opinions*	6 (16.2%)	16 (43.2%)	5 (13.5%)	6 (16.2%)	2 (5.4%)
I feel safe to raise my need for support	8 (21.6%)	13 (35.1%)	2 (5.4)	10 (27.0%)	2 (5.4%)

* Not all participants responded to this question.

#### 2.5.7 Working Relationally in a Bio‐Medically Dominated Clinical Context

Participants spoke about the ambiguous positioning of families and carers in the mental health system. Some participants stated that the bio‐medical model creates a knowledge hierarchy which marginalises the Family/Carer LE worker's expertise, with their roles positioned as adjacent to, rather than integrated within clinical teams. For instance, a survey respondent explained that ‘as an advanced carer peer [worker], I was the lone part‐time worker stretched across a service working within a hierarchical medical model’. Many workers described feeling that their LE knowledge was not seen as legitimate by colleagues, and they were questioned about their qualifications.

I feel within the service, there's a bit of elitism. Some of the clinicians would say What university, what qualifications do you have? And I find that quite insulting. And again, I've worked in [my previous role] for 23 years, and I found the lack of accountability within our service very confronting with judgement from clinicians.

[FGP19]

This type of questioning served to undermine the Family/Carer LE workers sense of value within the bio‐medical knowledge hierarchy.

#### 2.5.8 Isolation in the Workplace

Isolation in the workplace was a prominent theme in both the survey and focus groups. Thirty‐two percent either strongly agreed or agreed with the statement ‘I feel isolated in the workplace because of being a family/carer LE worker’. Almost a quarter of respondents reported that they were the only Family/Carer LE worker in a service (21.6%), with one survey participant stating they were the only LE staff of 600 employees. Limited staffing and part‐time hours further reduced opportunities for peer connection.

I have minimal interaction with, and feedback from, other Family/Carer peer workers as I am the sole person in this role at the place I work. There is minimal interaction with others in this role, except for a monthly carer network meeting and occasional support from my peer supervisor.

[Survey participant]

Those working in rural or remote areas faced additional challenges, often covering multiple sites alone. One worker reflected, ‘I work in a remote role, so am quite isolated at times, mirroring my lived experience as a caregiver, can feel under‐supported, at times’ [Survey participant]. These conditions contributed to a sense of disconnection and increased workload, with 46.8% of respondents indicating they had considered leaving or had left their roles. These precarious workplace conditions call into question the sustainability of this workforce.

#### 2.5.9 Moral Injury in the Workplace

Although 65.8% of respondents reported feeling safe at work, more than half (58.4%) had experienced moral distress. The distress was often related to witnessing how carers were treated by other staff. One participant illustrated this by highlighting how signs of carer burnout were often misinterpreted by clinicians, leading to misunderstanding and stigma:

We've often experienced carer burnout ourselves, but what can be that moral distressing situation is when clinicians or doctors will be judgmental about the carer and things like that they're not caring enough or their caring looks wrong and when you're sitting in that space and you're hearing from your work colleagues making judgemental conversations about a carer that can cause, like I said, that moral distress, you know, and some of those things might be simply a lack of understanding from their perspective.

[FGP 11]

Accessing support was another concern, with some workers feeling unsafe disclosing their distress, due to concerns about how it may be viewed.

There's far too much vulnerability, too many psychological hazards, and if you admit to vulnerability or being triggered, it can sometimes be used against you, that you are not up to this job. So instead of being supported, you're given the exact opposite.

[FGP5]

The survey results also demonstrated these concerns with the majority of survey participants (56.7%) indicating they do not feel safe to raise their need for support (see Table 2).

#### 2.5.10 Lack of Career Pathways and Professional Development

Family/Carer LE workers also frequently reported limited opportunities for professional development and career progression leaving them uncertain about career progression. One survey respondent expressed this concern:

There is no clear career pathway for carer peer workers. There is no opportunity for advancement or promotion. There is no opportunity for full‐time work.

[Survey participant]

The absence of career pathways was compounded by inconsistent access to training. Workers described receiving ‘one‐off training’ [FGP14] and having to seek out their own learning opportunities, often without financial or organisational support. This was due to ‘ongoing learning and training [being] seen as a low priority in comparison to clinician training’ [FGP10]. Workers explained how they accessed free training because ‘a lot of us don't have a budget for professional development and going to conferences cost, you know $600 is just not an option [FGP12]. A number of focus group participants noted that the Certificate IV in Mental health ‘is the only formal qualification at the moment that I am aware of and it's not a one size fits all’ [FGP9]. This lack of investment in growth not only affected workers' confidence but also their ability to contribute meaningfully within their teams. One survey respondent shared, ‘I feel like I'm not given the same opportunities to grow in my role as other staff. I'm not included in training or development opportunities’. These conditions contributed to a sense of stagnation and frustration, with many workers questioning the long‐term viability of their positions.

The precariousness of their roles, the lack of pay parity, role clarity, the feelings of being undervalued and unsupported and a lack of professional development and career pathways were all seen as significant barriers to retention and sustainability within the Family/Carer LE workforce.

#### 2.5.11 Supporting the Potential of the Family/Carer LE Workforce

##### 2.5.11.1 Organisational and Leadership Support

Supportive leadership from managers and others in the organisation was seen as a crucial factor in maintaining the wellbeing and recognition of Family/Carer LE workers. Survey data indicated just over half of respondents (51.4%) felt that feedback from their line manager helped them grow professionally, while 20.0% were neutral and 28.5% disagreed or strongly disagreed. A few focus group participants highlighted how advocacy from Family/Carer LE workers, both current and former, had led to meaningful cultural shifts within their organisations. This leadership support, however, was often described as fragile, hinging on the presence of specific individuals rather than being embedded across the organisation. One focus group member explained:

I'm in a psychologically safe workspace where my opinion is respected and everyone's opinions are respected, and I feel like I'm making a difference, then for me, that's all I need to keep me happy. And you know, I'm just one leadership change away from that changing, you know…what I've got now could change in an instant, you know, with just a change of programme manager. So it's scary.

[FGP12]

This quote clearly shows how organisational support can be unstable and unreliable.

#### 2.5.12 Supervision as a Vital Element

Reflective supervision was identified as a key support mechanism for Family/Carer LE workers. Seventy‐two percent of survey respondents reported having access to supervision which allowed them to reflect on their practice, integrate their LE into their work and process the impact of the work. Eighty percent of those who reported having access to supervision found this support helpful. Focus group participant 6 stated:

Supervision is about keeping the worker healthy and well, and this is forgotten. It is a necessity, not something that would be nice.

However, the quality and relevance of supervision varied. Many workers stressed the importance of receiving guidance from someone who is an experienced Family/Carer LE worker. One participant described the impact of such support as following:

It was with a very specific Family/Carer peer worker who had extensive understanding and knowledge … on how things could work within a team and how I could expand on my understanding of my peer practice. [It] … just made a huge difference to me and how I developed professionally. It was just fantastic.

[FGP5]

Supervision formats differed, with most respondents (64.9%) having received individual supervision, while 18.9% participated in group sessions. For those in isolated roles, group supervision, often called co‐reflection was especially valued. One participant explained,

We've just started a group. A carer peer supervision space, which has just started [by] linking in via Teams… So it's not as formal and structured as one‐on‐one [supervision], but it allows… some of those ‘aha' moments that you know you're understanding where somebody else is doing your role and you know I find that really so empowering because you can hear what somebody else is struggling with, how they've overcome it and then sort of bringing that into your own work.

[FGP10]

Given the various precarious conditions for Family/Carer LE workers, supervision was crucial to support them to navigate these workplace challenges.

#### 2.5.13 Professional Development and Career Progression

Participants indicated that professional development plays an important role in supporting the potential of the workforce. While workers appreciated the provision of professional development, only 25.7% indicated there was a clear pathway for professional development (see Table 4).

**Table 4:** Workplace professional developmental support.

Statement	Strongly agree	Agree	Neutral	Disagree	Strongly disagree
There is a clear pathway for professional development*	3 (8.1%)	6 (16.2%)	8 (21.6%)	10 (27.0%)	8 (21.6%)
I have received enough training to do my role*	7 (18.9%)	7 (18.9%)	8 (21.6%)	8 (21.6%)	5 (13.5%)
I am often asked to perform roles outside my job description*	7 (18.9%)	12 (32.4%)	8 (21.6%)	4 (10.8%)	4 (10.8%)
I have a good understanding of what I am required to do*	11 (29.7%)	17 (45.9%)	1 (2.7%)	2 (5.4%)	0
I have the necessary resources to do my job (e.g., office space, equipment)*	11 (29.7%)	12 (32.4%)	6 (16.2%)	3 (8.1%)	3 (8.1%)

* Not all participants responded to this question.

While the Family/Carer LE workforce was explicit about how organisational and leadership support, professional development and supervision were essential to sustaining themselves and help them progress in their careers, these results indicate a mixed experience in accessing these supports.

## 3 Discussion

This mixed methods study investigated the workplace experiences of the Family/Carer LE workforce in Queensland and extended the work of the Rising Together project conducted in Victoria. Like the Victorian Family/Carer LE workforce, the Queensland Family/Carer LE workforce is equally passionate about and committed to their roles (Sellick et al., 2024). This indicates the strong drive of Family/Carer LE workforce who, often motivated by their own experiences of navigating the mental health system are wanting to improve the experiences of other families and carers (Sellick et al., 2024). However, despite this commitment, the participants reported that their capacity to promote relational, family‐centred practices and advocate for systemic change were constrained by various structural challenges. Many worked in isolation and experienced marginalisation within the public and non‐government mental health sector. In many ways, the experiences of the Family/Carer LE workforce mirrors the carer experience of being unsupported and isolated [4, 5], frequently having to be the ‘squeaky wheel’ to be heard. The findings demonstrate how challenging it can be for this workforce to advocate for family inclusion within services that prioritise individual clinical care, where the needs of carers and families are often overlooked and/or pathologised. The prevalence of moral injury is of concern for this workforce, because witnessing families or carers being treated poorly by services is particularly distressing when LE workers have had similar life experiences, which can lead to burnout and workers leaving their role (Sellick et al., 2024). Additionally, moral injuries may be exacerbated when peer workers are confronted with practices that violate their values and they are rendered powerless to challenge them, even though advocacy is a fundamental part of their role [26].

Lack of role clarity was a key finding which demonstrates the precarious positioning of the Family/Carer LE workforce. Job descriptions provide a critical mechanism to support safety, and decent working conditions especially in workforces vulnerable to role ambiguity and precarity [35]. This basic workplace condition, however, was not a shared experience for this workforce, with our findings highlighting how written job descriptions continued to vary from the spoken and unspoken expectations of this workforce. Role ambiguity meant that in addition to supporting and advocating for family inclusion, Family/Carer LE workers were also tasked with legitimising their roles, educating colleagues, while striving to foster collaborative dynamics within multidisciplinary teams. Although some challenges, such as the geographical isolation experienced by those in rural and remote areas have not been explored in previous research, many of the structural vulnerabilities echo concerns raised in broader Family/Carer LE workforce literature [24, 30, 31]; Sellick et al., 2025).

In addition to the structural organisational barriers, findings illustrated precarious employment by the part‐time or casual workplace conditions and the lack of possibility for career progression. For many, this also meant little flexibility in their working hours which impacted on their caring responsibilities. It is possible that the part‐time work opportunities are a reflection on what organisations are willing to contribute. The prevalence of part‐time and casual positions in this workforce, however, aligns with broader patterns of women's employment, often driven by the need to balance work with unpaid caring responsibilities [36]. Women remain disproportionately represented in care‐related professions, contributing to a cycle of undervaluation and economic vulnerability [37]. This precarity, coupled with a lack of career progression for many Family/Carer LE roles undermines the retention and growth of this workforce. Similarly to the Consumer LE workforce, these conditions not only contribute to emotional fatigue, professional disenchantment and burnout [25, 38, 39] but also compromise Family/Carer LE workforce sustainability. This could potentially limit the sustainability and growth of this workforce as Family/Carer LE workers who cannot see viable opportunities for career development and may leave the sector.

Lastly, the findings illustrated how the unique emotional labour inherent within this work necessitates a careful consideration of support and supervision for LE workers. Ongoing reflective dialogue is needed to explore how Family/Carer LE workers navigate the contradictions of working relationally within a bio‐medical model to explore alternative ways to position themselves [40]. Participants believed it was critical to receive supervision directly from a staff member who had experience of being in a similar LE role. This is reflective of the broader LE literature that positions Family/Carer LE work as a specific professional discipline that requires specific LE supervision, along with tailored training and development opportunities [41, 42].

### 3.1 Limitations

While 37 valid responses were received, which were estimated to represent almost half of the Family/Carer LE workforce, the true size of the workforce is unknown and as such it is difficult to determine if the sample was representative. Furthermore, while survey participants were drawn from metropolitan, rural, and remote areas, the focus group participants were predominantly metropolitan. The study is a cross‐sectional design and only provides a single‐point snapshot and cannot capture changes over time. There is also the possibility of self‐selection bias, as those who chose to participate may differ in important ways from those who did not. Finally, while some aspects of diversity are acknowledged, because of the low numbers we were not able to fully explore how different aspects of identity, such as gender, cultural background, or disability status, intersect to shape individual experiences within the workforce.

### 3.2 Policy Implications

Embedding Family/Carer LE roles into mental health services requires structural and cultural change and a multifaceted approach is needed. The recommendations below align with the Queensland Framework for Mental Health Lived Experience Workforce Development [43] and the Victorian Carer Perspective Supervision Framework [42].

*To strengthen the Family/Carer LE discipline's identity* a clear scope of practice and aligning position descriptions to these are needed [41]. To ensure that the roles are fully integrated, organisational readiness is essential and can be fostered through training of mental health staff about the unique contributions of Family/Carer LE roles and relational recovery approaches. Research funding to understand and evaluate the impact of Family/Carer LE roles would also enhance role clarity.

*To support the wellbeing and sustainability of the workforce* access peer co‐reflection spaces, and reflective supervision led by experiences Family/Carer LE supervisors, as well as the development of supervisory capacity within this workforce are essential. This supervision needs to be consistent, safe, reflective, and grounded in Family/Carer LE values.

*To build leadership and career structures within the workforce* it is critical that Family/Carer LE workers are employed in leadership and governance roles as well as the creation of clear career pathways. It is equally important to address the employment precarity with strategies such as fair remuneration, dedicated LE classification streams, and recognition within industrial frameworks, as well as, initiatives that cultivate inclusive environments through flexible work arrangements for staff with caregiving responsibilities.

Together, these strategies form a comprehensive framework for embedding Family/Carer LE roles into mental health systems in a sustainable and impactful way.

## 4 Conclusion

This study provides a comprehensive overview of the experiences of Family/Carer LE workforce in Queensland's mental health system, highlighting both the value this workforce brings and the persistent challenges they face. The Family/Carer LE workforce plays a vital role in supporting families and carers, bridging gaps in mental health services, and advocating for family‐inclusive practices. Their unique perspective and expertise offer invaluable insights that can enhance the quality and effectiveness of mental health care. While this is the case, structural and cultural limitations continue to hinder the full integration and recognition of this workforce. Achieving meaningful reform in family‐inclusive mental health care requires collective action and monetary investment. Change cannot rest solely on the shoulders of Family/Carer LE workers. These precarious work conditions underscore the urgent need for systemic change, including the integration of family inclusive practices and relational recovery approaches into mental health care. This study underscores the need for all stakeholders to move beyond the current ‘squeaky wheel’ paradigm and create an environment where this workforce can thrive and fully contribute to the wellbeing of families and carers across Queensland. By doing so, we can build a more responsive, compassionate, and effective mental health system that honours the essential role of families and carers and affirms their right to support and wellbeing.

## Author Contributions

**Katherine Reid:** conceptualisation, investigation, funding acquisition, writing – original draft, methodology, validation, writing – review and editing, visualisation, formal analysis, project administration, data curation, supervision, resources. **Lorna Downes:** conceptualisation, investigation, writing – review and editing, writing – original draft, funding acquisition, software, formal analysis. **Victoria Stewart:** conceptualisation, funding acquisition, writing – original draft, methodology, writing – review and editing, software, formal analysis. **Kath Sellick:** conceptualisation, investigation, funding acquisition, writing – review and editing, methodology, visualisation. **Marianne Wyder:** conceptualisation, investigation, funding acquisition, writing – original draft, methodology, validation, visualisation, writing – review and editing.

## Conflicts of Interest

The authors declare no conflicts of interest.

## Supporting information

Supporting File

## Data Availability

The data that support the findings of this study are available on request from the corresponding author. The data are not publicly available due to privacy or ethical restrictions. Data have been collected via an online survey and focus groups. Due to the potential to identify specific Family/Carer LE workers in the data, these data are not publicly available.
