# Supplementary material for: Beyond ‘The Squeaky Wheel’: The Passion, Precarity and Potential of the Family/Carer Lived Experience Workforce in the Queensland Mental Health System
Source: Health Expect. 2026 Jun 8;29(3):e70720. doi: 10.1111/hex.70720 (PMC13244664; doi:10.1111/hex.70720)
Supplement: Supplementary file 1 — Supporting File [file HEX-29-e70720-s001.docx]

# Eligibility criteria

1. Have you worked in Queensland?

Yes – go to next question

No - go to end of survey for ineligible participants

1. Have you worked in the emotional health and well-being/mental health sector in Queensland in the last 5 years?

Yes – go to next question

No - go to end of survey for ineligible participants

1. Was family/carer lived experience an essential requirement of your role (i.e. you are asked to use your family/carer lived experience in your work)

Yes – go to next question

No - go to end of survey for ineligible participants

1. How recent was your employment in a family/carer lived experience (in paid, unpaid voluntary or paid voluntary) role?

- Currently employed in a family/carer role (next question)
- Not currently employed in a family/carer role but have been in the last 5 years (next question)
- Not currently employed in a family/carer role but was employed in a role more than 5 years ago (go to end of survey for ineligible participants)

# Demographics

### **Demographics: Diversity in workforce**

We are interested in the diversity of the family/carer workforce. The following questions relate to information about your yourself and your identity.

1. What is your age?

- 0- 17 (redirected to the end of the survey for ineligible participants)
- 18-29
- 30-39
- 40-49
- 50-59
- 60-69
- 70-79
- 80+
- Rather not say

1. What gender do you identify as?

- Male
- Female
- Non-binary/third gender
- Self describe (free text space to self-describe)
- Prefer not to say

1. Do you identify as (tick as many as apply):

- Self-describe (free text to self-describe)
- Aboriginal
- Torres Strait Islander
- Culturally and linguistically diverse
- Refugee or asylum seeker
- Lived/living experience as a carer
- LGBTIQA+
- Living with psychological distress
- Living with a disability
- Have lived or living experiences as a consumer of mental health services
- Prefer not to say
- Not applicable

1. What is the highest level of education you have completed so far?

·         Secondary school (redirect to question 10)

·         Vocational education certificate (e.g. Certificate III, Certificate IV)

·         Vocational education diploma/advanced diploma

·         Undergraduate tertiary qualification

·         Postgraduate tertiary qualification

·         Other (please specify)  (redirect to question 10)

1. What kind of qualification(s) do you hold? (eg: Bachelor of Arts, Masters of Social Work, Cert IV Mental Health Peer Work) (open ended)
2. What is your lived experience of mental health caregiving or support (tick as many as apply)?

- Caring for child(ren) under 18 years
- Caring for adult child(ren)
- Caring for grandchild/ren) under 18 years
- Caring for adult grandchild(ren)
- Caring for partner(s) or spouse(s)
- Caring for sibling(s)
- Caring for parent(s)
- Caring for friend(s)
- I’d rather not say
- Other (please specify

1. Have you also worked in the emotional health and well-being/mental health sector in a non-family carer lived experience role? (for example, allied health role)
   - Yes- (go to question 12)
   - No (go to question 13)
2. Please described your previous non-lived experience role? (open ended)
3. How long have you worked in a family/carer lived experience role?

- Less than a year
- 1-2 years
- 3-5 years
- 6-10 years
- Over 10 years

1. While working in a family/carer lived experience role, have you held more than one role concurrently?

Yes (go to question 15)

No (go to question 16)

### New section

### **Demographics- Family/carer lived experience role**

### How many family/carer lived experience roles have you had in the last years?

1

2

3

4+

### If you have worked in more than one position, please select one family carer lived experience role that you are currently working in or have recently worked in. If you are a peer worker and also in a representative role, you can complete this peer worker survey, and then complete a shorter representative role survey.

1. Are you currently working in this role?

Yes

No

1. When did you commence this role?

In the last 6 months

In the last year

In the last two years

Between two and five years ago

Over five years ago

1. Is family/carer lived experience in your role title or position description?

Yes

No

1. Which role best describes your family/carer lived experience role?

Coordinator/manager

Family/Carer Consultant

Family/Carer Facilitator

Family/Carer Representative (on committees, projects, advisory groups etc) **(skip to question 89)**

Educator/trainer

Family/Carer peer worker

Senior Family/Carer peer coordinator

Family/ Carer peer worker

Peer group facilitator

Researcher

Other (please specify)

1. Where is this role located?

- Metropolitan centre
- Rural (town or city population over 10,000 people)
- Remote (town or city population less than 10,000 people)
- Statewide service
- National service
- Other Please specify (open-ended)

1. What part of the mental health sector is this position in?

· Publicly funded mental health and health service

· Private mental health service

· Not for Profit/Community Managed Mental Health Service (e.g. Mind, Wellways)

· Other (please specify)

1. Who do you work with (tick which apply)?

- Child and Youth Mental Health
- Adult mental health
- Alcohol and other drugs
- Older persons mental health
- Service-wide
- Other (please specify)

1. Do you work in inpatient or community settings?

- Inpatient
- Community
- Both
- Other (please specify)

1. What type of employment do you have in this role?

Full time

Part time

Permanent

Casual

On fixed-term contract

Unpaid voluntary

Paid voluntary

Other - please specify (How many hours did/do you typically work in this role per fortnight? (open ended)

1. Would you like to vary your working hours?
   - Yes, I would like to work more
   - Yes, I would like to work less
   - No
   - Unsure
2. What is/was your annual average income (before tax) in your family/carer lived experience role?

- $0
- $1-$10,399
- $10,400-$15,599
- $15,600-$20,799
- $20,800- $31,199
- $31,200-$41,599
- $41,600- $51,999
- $52,000-$64,999
- $65,000-$77,999
- $78,000-$103,999
- $104,000 or more
- I don’t know my annual rate, my hourly rate is (please specify)
- I’d rather not say

1. Do you do unpaid or out of hours work related to your family/carer lived experience work? (tick all that apply)

- I don’t do any unpaid or out of hours work
- I read and review documents outside of work hours
- Learning more about systems, policies etc
- Attending meeting or consultations that fall outside my paid hours
- Attend meetings my workplace does not pay me to attend
- Checking and responding to emails
- To keep on top of my workload or complete my required tasks
- Reflective practice (external supervision, journaling)
- Training that you see as essential to your role
- Other (please specify)

1. On average, how many hours a fortnight do you do this unpaid work?

0-2 hours

2-5 hours

5-10 hours

10-20 hours

Over 20 hours

1. How many other family/carer lived experience workers are employed in your service?

- 0
- 1
- 2
- 3-5
- 6-10
- 11-20
- Over 20

1. How frequently do you have contact with family/carer lived experience workers in your organisation?

- No contact
- daily contact
- weekly
- monthly
- less than monthly
- Not applicable

1. I am content with the level of contact I have with other family/carer lived experience workers

Strongly agree

Agree

Neither agree nor disagree

Strongly disagree

1. Thinking about your responses to the questions about contact with other family/carer lived experience workers, is there anything further you would like to say? (optional)

Workplace Experiences

The following questions relate to your experiences as a family/carer lived experience worker. Please rate to what degree you agree with the following statements when you think about your role.

1. I am valued by the families and carers that I support in my role

- Strongly Agree
- Agree
- Neutral
- Disagree
- Strongly Disagree
- Not applicable - I don’t directly support families and carers in my role

1. I make a difference to the families and carers

- Strongly Agree
- Agree
- Neutral
- Disagree
- Strongly Disagree

1. I am understood by my family/carer worker lived experience colleagues

• Strongly Agree

• Agree

• Neutral

• Disagree

• Strongly Disagree

• Not applicable

1. I am valued by my family/carer worker lived experience colleagues

• Strongly Agree

• Agree

• Neutral

• Disagree

• Strongly Disagree

- Not applicable

1. I am supported by my family/carer worker lived experience colleagues

Strongly Agree

• Agree

• Neutral

• Disagree

• Strongly Disagree

• Not applicable

Please comment on your reasons for your rating of the last three questions (optional) (open text)

1. I am understood by my consumer lived experience worker colleagues

• Strongly Agree

• Agree

• Neutral

• Disagree

• Strongly Disagree

• Not applicable

1. I am valued by my consumer lived experience worker colleagues

• Strongly Agree

• Agree

• Neutral

• Disagree

• Strongly Disagree

• Not applicable

1. I am supported by my consumer lived experience worker colleagues

• Strongly Agree

• Agree

• Neutral

• Disagree

• Strongly Disagree

• Not applicable

Please comment on your reasons for your rating of the last three questions (optional) (open text)

1. I am understood by non-lived experience worker colleagues (e.g. clinicians, nurses, social workers, support workers)

• Strongly Agree

• Agree

• Neutral

• Disagree

• Strongly Disagree

• Not applicable

1. I am valued by non-lived experience worker colleagues (e.g. clinicians, nurses, social workers, support workers)

• Strongly Agree

• Agree

• Neutral

• Disagree

• Strongly Disagree

• Not applicable

1. I am supported by non-lived experience worker colleagues (e.g. clinicians, nurses, social workers, support workers)

• Strongly Agree

• Agree

• Neutral

• Disagree

• Strongly Disagree

• Not applicable

Please comment on your reasons for your rating of the last three questions (optional) (open text)

1. I am understood by my line manager

• Strongly Agree

• Agree

• Neutral

• Disagree

• Strongly Disagree

- I don’t have a line manager

1. I am valued by my line manager

• Strongly Agree

• Agree

• Neutral

• Disagree

• Strongly Disagree

I don’t have a line manager

1. I am supported by my line manager

• Strongly Agree

• Agree

• Neutral

• Disagree

• Strongly Disagree

I don’t have a line manager

1. My role is understood within my organisation

• Strongly Agree

• Agree

• Neutral

• Disagree

• Strongly Disagree

1. My role is valued within my organisation

• Strongly Agree

• Agree

• Neutral

• Disagree

• Strongly Disagree

1. I am supported by my organisation

• Strongly Agree

• Agree

• Neutral

• Disagree

• Strongly Disagree

I am satisfied in the work that I do as a family/carer lived experience worker

• Strongly Agree

• Agree

• Neutral

• Disagree

• Strongly Disagree

**Inclusion within the Team and Organisation**

The following questions relate to your experiences as a family/carer lived experience worker. Please rate to what degree you agree with the following statements when you think about your position.

1. The work I do with families and carers integrates well with the core purpose of the organisation

- Strongly Agree
- Agree
- Neutral
- Disagree
- Strongly Disagree

1. I feel part of a team in my workplace

- Strong Agree
- Agree
- Neutral
- Disagree
- Strongly Disagree
- I don’t work in a team

1. I feel excluded in the workplace because of being a family/carer lived experience worker

- Strongly Agree
- Agree
- Neutral
- Disagree
- Strongly Disagree

1. I feel isolated in my workplace because of being a family/carer lived experience worker

- Strongly Agree
- Agree
- Neutral
- Disagree
- Strongly Disagree

1. I feel safe in the workplace

• Strongly Agree

• Agree

• Neutral

• Disagree

• Strongly Disagree

Please comment on your reasons for your rating of this question (open text)

1. I am free to express my opinions in the workplace

• Strongly Agree

• Agree

• Neutral

• Disagree

• Strongly Disagree

1. I feel ignored or dismissed by colleagues because I work in a family/carer lived experience role

• Strongly Agree

• Agree

• Neutral

• Disagree

• Strongly Disagree

1. I feel safe to raise my need for support

• Strongly Agree

• Agree

• Neutral

• Disagree

• Strongly Disagree

Please comment on your reasons for your rating of this question (open text)

1. Have you experienced any of the following: please tick all that apply (optional)

I have witnessed or seen evidence of family/carers being dismissed

I have witnessed or seen evidence of discriminatory treatment of consumers

I have witnessed or seen evidence of discriminatory treatment of family/carers

I have witnessed or seen evidence of disregard for the recovery goals of the consumer

I have witnessed or seen evidence of disregard for the wellbeing of families and carers

I have witnessed or seen evidence of restrictive practices

Other (please specify)

1. I have experienced moral distress in the workplace (Moral distress is when a person is exposed to events that are morally compromising and go against deeply held beliefs and values)

• Strongly Agree

• Agree

• Neutral

• Disagree

• Strongly Disagree

1. I am paid adequately for my work as a family/carer lived experience worker

• Strongly Agree

• Agree

• Neutral

• Disagree

• Strongly Disagree

1. Do you feel that the conditions of your employment are different to that of your non-lived experience colleagues? (For example, conditions such as the resources you can access to do your role)

- Yes
- No

1. If Yes, tick as many as apply

- I only have part-time or casual positions available to me
- No permanent full-time positions are available
- I am not allowed within certain areas of the workplace
- I am employed on a fixed-term contract
- I get paid less than my non-lived experience colleagues of similar skill level or experience
- I don’t have study leave or have to negotiate to take study leave
- I have access to flexible working arrangements due to my caring role
- I do not have access to training opportunities
- I have minimal opportunities for career advancement
- Other, please specify (open text)

Recruitment and appointment processes for family/carer lived experience roles are fair?

• Strongly Agree

• Agree

• Neutral

• Disagree

• Strongly Disagree

1. Are there any comments you would like to make in relation to your rights as a working carer (e.g. requesting flexible work arrangements, carers leave) (open ended)

### **Workplace support and supervision**

The following two questions relate to your line management in your role.

Line management involves the oversight and guidance of the direct operational activities of the staff member. This includes workloads, allocation of tasks, contracts, leave, human resource issues, performance development and the overseeing of compliance with policy and targets.

1. The professional background of your line manager best aligns with:

- Family/carer lived experience discipline
- Consumer lived experience discipline
- Nursing
- Social Work
- Occupational Therapy
- Psychology
- Medicine/psychiatry
- Other (Please specify)
- Don’t know

1. I get given feedback from my line manager that enables me to grow in my profession

- Strongly Agree
- Agree
- Neutral
- Disagree
- Strongly Disagree

1. How often do you have contact with your line manager about your work?

Daily

Weekly

Fortnightly

Monthly

A few times a year

Other (please specify)

The following questions relate to reflective supervision, including family/carer discipline specific supervision, peer supervision and co-reflection.

Supervision focuses on reflective practice, the impact of your work, debriefing, and the application of your skills and lived experience in your work. It should be a safe space to explore strengths and strategies as well as problem solving issues, challenges, and tensions.

1. Do you receive supervision in your role?

Yes

No (go to question 74)

1. Who provides your primary reflective supervision (Select one)

- Reflective supervision, provided by my line manager (additional to line management supervision)(not line management supervision)
- Reflective supervision provided by another staff member (from a different discipline) in the organisation
- Reflective supervision provided by family/carer lived experience worker within my organisation
- External supervision, paid for by my organisation
- External supervision, I pay for myself
- Other, please specify
- I am not provided with any reflective supervision

1. What kind of reflective supervision is provided (tick all that apply)

Individual

Group Supervision

1. How often do you access reflective supervision?

Daily

Weekly

Fortnightly

Monthly

A few times a year

Other (please specify)

1. Please rate to what degree you find the reflective supervision helpful

- Very helpful
- Helpful
- Neutral
- Unhelpful
- Very helpful

1. I have had the following offered to me to support my learning in this role (tick as many as apply):

- I have received relevant training within my organisation
- I have been granted study leave to pursue further training (paid/unpaid) outside of my organisation
- My employer has funded further training
- My employer has paid for conference attendance
- I have had access to scholarships/grants to further my training
- Other (please specify)
- I have not received any support for my learning in this role

1. Do you access any other forms of support to enable you to thrive in your position?

Yes, please specify (open text)

No

1. Please share any additional comments about support?

Please rate the following statements.

1. As a family/carer lived experience worker there is a clear pathway for professional development

- Strongly Agree
- Agree
- Neutral
- Disagree
- Strongly Disagree

1. I have received enough training to allow me to fulfill my role to the best of my ability.

Strongly Agree

Agree

Neutral

Disagree

Strongly Disagree

1. I am often asked to perform roles outside of my job description

Strongly Agree

Agree

Neutral

Disagree

Strongly Disagree

1. I have a good understanding of what I am required to do in my role

Strongly Agree

Agree

Neutral

Disagree

Strongly Disagree

1. I have the necessary resources to do my job (eg: phone, office space, equipment)

Strongly Agree

Agree

Neutral

Disagree

Strongly Disagree

1. Any additional comments about resources that would enable you to do your job? (open text)
2. Have you left, or considered leaving the family/carer lived experience role you have chosen to tell us about?

- I have left my family/carer lived experience role
- I have considered leaving my family/carer lived experience role
- No (redirect to question ..)

1. Why did you leave or consider leaving? (tick all that apply)

- I found another more suitable family/carer role
- I found a consumer role
- I found another non-lived experience role in the mental health field
- I found another role outside of the mental health field
- I felt unsafe in the workplace
- I felt unvalued in the workplace
- I felt unsupported in the workplace
- I felt that I was not paid enough
- I needed a job with more hours
- My role was made redundant
- I decided to retire
- My contract ended
- Other (please specify)

**Open ended**

1. You have answered this survey in relation to a specific role. If you have or have had other family/Carer lived experience roles, do you have any comments (negative or positive) about your other roles? (open text)
2. What advice would you give to an organisation who is planning on employing family/carer lived experience workers?
3. Is there anything else you would like to tell us about your experience as a family/carer lived experience worker? (open-ended)
